# Hotspot mutation profiles of AKT1 in Asian women with breast and endometrial cancers

**DOI:** 10.1186/s12885-021-08869-3

**Published:** 2021-10-21

**Authors:** Tatsunori Shimoi, Jun Hashimoto, Kazuki Sudo, Akihiko Shimomura, Emi Noguchi, Chikako Shimizu, Mayu Yunokawa, Kan Yonemori, Hiroshi Yoshida, Masayuki Yoshida, Tomoyasu Kato, Takayuki Kinoshita, Takahiro Fukuda, Yasuhiro Fujiwara, Kenji Tamura

**Affiliations:** 1 Department of Medical Oncology, National Cancer Center Hospital, 5-1-1 Tsukiji, Chuo-ku, Tokyo, 104-0045 Japan; 2 Course of Advanced Clinical Research of Cancer, Juntendo University Graduate School of Medicine, 3-1-3 Hongoh, Bunkyo-ku, Tokyo, 113-0033 Japan; 3 Division of Medical Oncology, Department of Internal Medicine, St. Lukes International Hospital, Tokyo, Japan; 4 Department of Breast and Medical Oncology, National Center for Global Health and Medicine, Tokyo, Japan; 5 Department of Gynecologic Oncology, Cancer Institute Hospital of Japanese Foundation for Cancer Research, Tokyo, Japan; 6 Department of Diagnostic Pathology, National Cancer Center Hospital, Tokyo, Japan; 7 Department of Gynecology, National Cancer Center Hospital, Tokyo, Japan; 8 Department of Breast Surgery, National Cancer Center Hospital, Tokyo, Japan; 9 Division of Breast Surgery, Tokyo Medical Center, Tokyo, Japan; 10 Department of Hematopoietic Stem Cell Transplantation, National Cancer Center Hospital, Tokyo, Japan; 11 Innovative Cancer Center, Department of Medical Oncology, Shimane University Hospital, Shimane, Japan

**Keywords:** *AKT1* mutation, Breast cancer, Endometrioid histology, HER2, Endometrial cancer

## Abstract

**Background:**

The V-Akt murine thymoma viral oncogene (*AKT*) *1* (E17K) is a subfamily of serine/threonine protein kinases that affects the survival, proliferation, and invasion of cancer cells. The clinicopathological features and frequencies in Asian populations with *AKT1* mutations in breast and endometrial cancers are unclear. Hence, we aimed to determine the frequencies and relationships between clinicopathological features and *AKT1* mutations in Asian women with cancer.

**Methods:**

We extracted DNA from 311 and 143 samples derived from patients with breast and endometrial cancers to detect the *AKT1* point mutation (hotspot), E17K. We examined correlations between clinicopathological features and *AKT1* mutation status.

**Results:**

The frequency of *AKT1* mutations in breast cancer was 7.4%, and they were found more frequently in human epidermal growth factor receptor 2 (HER2)-negative breast cancer subtypes, although this was not statistically significant (*P* = 0.08). The frequency of *AKT1* mutations in endometrial cancer was 4.1%, and the mutations were histologically detected only in endometrioid types. However, *AKT1* mutations did not correlate with relapse-free or overall survival of patients with breast or endometrial cancer.

**Conclusions:**

*AKT1* mutations are associated with HER2-negative subtype in breast cancer and in endometrial cancer with endometrioid histology. The frequencies of *AKT1* mutations in breast and endometrial cancers were similar between Asian and other regional women. The frequency of mutations is too low in both tumor types to talk about predictive significance.

**Supplementary Information:**

The online version contains supplementary material available at 10.1186/s12885-021-08869-3.

## Background

The phosphatidylinositol 3-kinase (PI3K)/v-Akt murine thymoma viral oncogene (AKT)/mTOR pathway is activated across many cancer lineages, and it is associated with cancer development and metastasis [1]. Approximately 70% of patients with breast cancer have activated phosphatidylinositol 3-kinase (PI3K). A mutation in PI3K catalytic subunit α is one of the most frequently activated in some cancers [2].

The *AKT1* subfamily of serine/threonine protein kinases affects cell survival, proliferation, and invasion*.* A dominant hotspot mutation is a glutamate 17 to lysine (E17K) substitution, and *AKT1* pleckstrin homology domain mutations activate serine-threonine kinase. Moreover, the findings of recent clinical trials have suggested that mutated *AKT1* is a potent predictive marker of responses to AKT inhibitors [3, 4].

A large-scale retrospective analysis of recent data derived from ~ 20,000 solid tumours revealed an *AKT1* mutation frequency of ~ 1% [1]. The reported frequencies of *AKT1* mutations are between 1.4 and 8.2% [5, 6] in breast cancer and 2.2 and 4.1% in endometrial cancer [7, 8].

However, the clinicopathological features and mutation frequency of *AKT1* in Asian populations with breast and endometrial cancers have not been examined. Here, we investigated the frequencies of *AKT1* mutations and their associations with clinicopathological features in Asian women with breast and endometrial cancers.

## Materials and methods

The pathological and clinical data of the cases used in this study were collected from the medical records in a retrospective manner.

### Tissue samples and patient selection

#### Breast cancer cohort

We obtained 118 frozen tumour surgical specimens and 193 formalin-fixed paraffin-embedded (FFPE) surgical or biopsy tissues that had been preserved in a biobank at the National Cancer Centre Hospital between May 1989 and December 2012. We extracted DNA from the frozen tumour specimens and FFPE tissues using QIAamp DNA Mini Kits and QIAamp DNA FFPE Tissue Kits, respectively (Qiagen GmbH, Hilden, Germany) as described by the manufacturer [9]. Mutations at the *AKT1* hotspot detected using peptide nucleic acid-locked nucleic acid (PNA-LNA) clamp methods in samples collected and stored at the National Cancer Center Hospital were analysed by LSI Medience Corporation (Tokyo, Japan) as described previously [10].

#### Endometrial cancer cohort

We extracted DNA from 142 DNA FFPE surgical tissue samples that had been preserved at the National Cancer Centre Hospital between May 1989 and November 2012 using QIAamp DNA FFPE Tissue Kits (Qiagen) as described by the manufacturer [9]. We detected the *AKT1* hotspot mutation using real-time PCR as described [8] with the forward and reverse primer sequences of exon 4 of *AKT1* (5′-CACACCCAGTTCCTGCCTG-3′ and 5′-CCTGGTGGGCAAAGAGGGCT-3′, respectively). The PCR cycling program comprised activation at 94 °C for 5 min, followed by 35 cycles at 94 °C for 30 s, 55 °C for 30 s, 72 °C for 60 s, and 72 °C for 10 min. The PCR products were sequenced using the BigDye terminator method and an auto-sequencer (Applied Biosystems, Foster City, CA, USA).

#### Histopathological evaluation

Oestrogen (ER) or progesterone (PgR) receptors were classified as positive or negative based on ASCO/CAP Guideline [11]. In all cases, at least 1% of cells staining was considered positive according to the criteria. Human epidermal growth factor receptor-2 (HER2) positivity was classified according to the guidelines of the American Society of Clinical Oncology/College of American Pathologists [12]. Hormone receptor-positive status was defined as positive for either ER or PgR. The histological and nuclear grade of breast cancer and endometrioid uterine carcinoma were graded as described in previous studies [9, 13] and in a previous report [14], respectively. Pathological evaluations are always performed by at least two expert pathologists (including HY and MY), although the results of diagnoses in institutional practice are used. ND in our Tables is because there was a time when it was not evaluated in the old days, and we could not re-evaluate it sufficiently because of the deterioration of pathological specimens.

#### Clinical outcome evaluation based on AKT1 mutation

Differences in outcomes between patients with and without *AKT1* mutations were assessed using data from 311 and 143 patients in breast and endometrial cancer subgroups. Differences between proportions for categorical variables were analysed using chi-square tests. Postoperative relapse-free (RFS) and overall (OS) survival was analysed using Kaplan–Meier curves. We defined RFS as elapsed time between the day of surgery and disease progression or the last follow-up for operable lesions. We defined OS as elapsed time between the first day of diagnosis and the date of death or the last follow-up. Significant prognostic factors associated with RFS and OS were determined by multivariate analysis using Cox hazard models. Clinically relevant factors for breast (initial stage, histologic grade, ER status, PgR status, HER2 status, and *AKT1* mutation) and endometrial (diagnostic stage, lymphovascular invasion, and *AKT1* mutations) cancers were included as covariates in multiple Cox proportional hazards models. All data were statistically analysed using JMP version 11 (SAS Institute, Cary, NC, USA). Statistical significance was set at *P* < 0.05.

## Results

### Prevalence of AKT1 hotspot mutation (E17K) in patients with breast cancer

The overall prevalence of *AKT1* mutations was 23 (7.4%) of 311 breast cancer samples.

### Association of AKT1 mutation with clinicopathological parameters in breast cancer

Table 1 shows the clinicopathological parameters according to *AKT1* mutations in the breast cancer samples. Mutated *AKT1* was not associated with prevalence, age, histologic subtype, initial stage (7th edition of UICC [the Union for International Cancer Control] TNM stage), tumour grade, lymphovascular invasion, and hormone receptor status. In the 16 patients with the so-called triple negative subtype, which is negative for both ER/PgR/HER2, two patients had AKT1 hotspot mutations. The number of patients with *AKT1* hotspot mutation was 13% (3/22). On the other hand, HER2-negative status tended to have more *AKT1* gene mutations, although this was not statistically significant (*P* = 0.08).

**Table 1:** Characteristics of Asian patients with breast cancer (*N* = 311)

	Total	*AKT1* mutation status		p
	N = 311 (%)	Wild-type *n* = 288 (%)	Mutated *n* = 23 (%)	
Median age (range; y)	52 (22–90)	52 (22–90)	48 (32–67	0.22
Initial TNM Stage				0.83
IA	71	66 (24)	5 (24)	
IB	2	2 (1)	0 (0)	
IIA	68	65 (23)	3 (14)	
IIB	59	53 (19)	5 (24)	
IIIA	46	42 (15)	4 (19)	
IIIB	12	10 (4)	2 (10)	
IIIC	16	15 (5)	1 (5)	
IV	28	27 (10)	1 (5)	
ND	10	8 (4)	2 (9)	
Histology				0.17
IDC	284	266 (92)	18 (78)	
ILC	11	9 (3)	2 (9)	
ND	16	13 (5)	3 (13)	
Grade				0.88
1	31	29 (10)	2 (9)	
2	146	135 (47)	11 (48)	
3	116	109 (38)	7 (30)	
ND	18	15 (5)	3 (13)	
Lymphovascular invasion				0.15
Positive	130	117 (41)	13 (57)	
Negative	121	115 (40)	6 (26)	
ND	60	56 (19)	4 (17)	
Hormone Receptor				0.94
Positive	264	249 (86)	20 (87)	
Negative	47	39 (14)	3 (13)	
HER2 status				0.08
Positive	55	54 (19)	1 (4)	
Negative*	256	234 (81)	22 (96)	

Bp, partial mastectomy; Bt, total mastectomy; HER2, human epidermal growth factor receptor-2; HR, hormone receptor; IDC, invasive ductal carcinoma; ILC, invasive lobular carcinoma; LVI, lymphovascular invasion; mut, mutation; ND, no data. * HER2 negative includes triple negative and ER+/PR+/HER2- cancers. Differences between proportions for categorical variables were analysed using chi-square tests.

The median follow-up duration was 73 (range 2.5–450) months. Twenty-eight patients initially had metastases, 148 of the 283 patients who initially had early breast cancer relapsed (137 and 11 with wild-type *and AKT1* mutations, respectively), and 48 died of breast cancer.

**Fig. 1:**
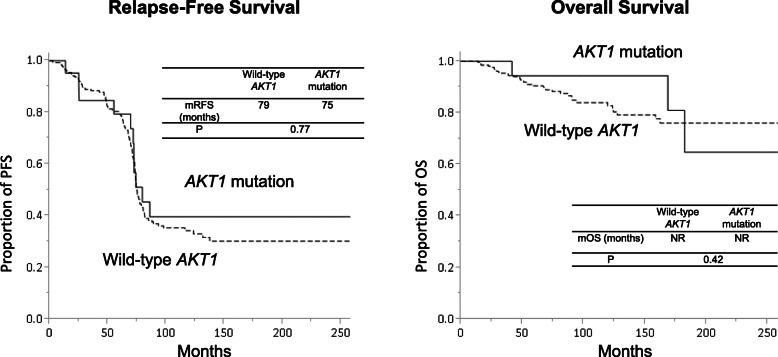
Relapse-free survival (RFS) and overall survival (OS) in patients with breast cancer. There were no significant differences in RFS and OS between patients with wild-type *AKT1* breast cancer and those with *AKT1*-mutated breast cancer

The RFS and OS did not significantly differ between patients with wild-type and mutated *AKT1* (Fig. 1). The median relapse-free durations in patients with mutated and wild-type *AKT1* were 75 and 79 months, respectively (*P* = 0.77). Multivariate analysis associated only the initial stage with OS (Additional Table 1).

### Prevalence of AKT1 hotspot mutations (E17K) in patients with endometrial cancer

The overall prevalence of mutated *AKT1* in *endometrial* cancer samples was 6 (4.1%) of 143 (Additional Figure 1).

### Associations between AKT1 mutation with clinicopathological parameters in endometrial cancer

Table 2 shows the clinicopathological parameters according to *AKT1* mutations in the *endometrial* cancer samples. Mutated *AKT1* was not associated with prevalence and age, histological subtype, initial stage (UICC 8th ed. /FIGO 2018), tumour grade in the histological subtype, or lymphovascular invasion.

**Table 2:** Characteristics of Asian patients with endometrial cancer (*N* = 143)

	Total	*AKT1* mutation status		p
	N = 143	Wild-type *N* = 137 (%)	Mutated *N* = 6 (%)	
Median age (range; y)	56 (28–89)	56 (28–89)	49 (44–62)	0.19
Initial TNM Stage				0.88
IA	54	52 (38)	2 (33)	
IB	27	25 (18)	2 (33)	
II	19	19 (14)	0 (0)	
IIIA	27	16 (12)	1 (17)	
IIIB	1	1 (1)	0 (0)	
IIIC	19	18 (13)	1 (17)	
IVB	6	6 (4)	0 (0)	
Histology				0.99
Endometrioid	131	125 (91)	6 (100)	
Serous	3	3 (2)	0 (0)	
Clear	2	2 (2)	0 (0)	
Carcinosarcoma	3	3 (2)	0 (0)	
Others	4	4 (3)	0 (0)	
Grade in Endometrioid histology				0.33
1	85	80 (58)	5 (83)	
2	26	25 (18)	1 (17)	
3	20	20 (15)	0 (0)	
Lymphovascular invasion				0.69
Positive	18	62 (45)	2 (33)	
Negative	125	75 (55)	4 (67)	

Differences between proportions for categorical variables were analysed using chi-square tests.

The median follow-up duration was 60 (range 0.6–275) months. Six patients initially had metastatic lesions, 18 of the 137 patients who initially had early endometrial cancer, relapsed (16 and 2 patients with wild-type *and* mutated *AKT1*, respectively), and 21 patients died of any cause. Three deaths were related to other types of cancer or other diseases, and 18 were due to endometrial cancer.

**Fig. 2:**
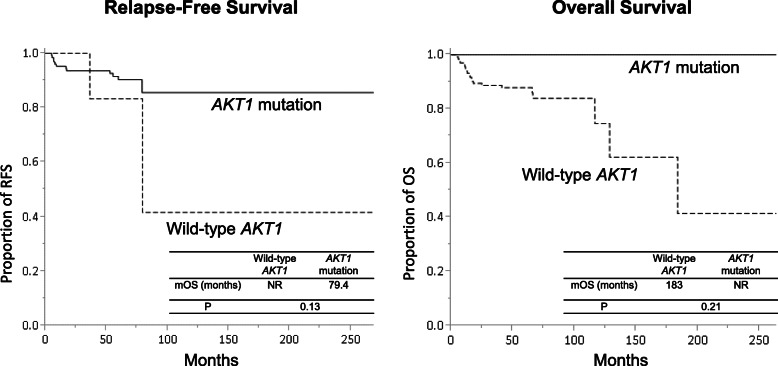
Relapse-free survival (RFS) and overall survival (OS) in patients with endometrial cancer. There were no significant differences in RFS and OS between patients with wild-type *AKT1* endometrial cancer and those with *AKT1*-mutated endometrial cancer

The RFS and OS did not significantly differ between patients with wild-type and mutated *AKT1* (Fig. 2). The median RFS in patients with mutated and wild-type *AKT1* were 77.8 months and not reached, respectively (*P* = 0.13). The median OS in patients with mutated and wild-type *AKT1* was not reached and 183 months, respectively (*P* = 0.21). Multivariate analysis associated the initial stage and AKT1 mutation with OS (Additional Table 2).

## Discussion

This is the first report of an association between clinicopathological features and *AKT1* mutation status in women with breast and endometrial cancers in Asia.

In the present study, we found *AKT1* mutations in 7.3 and 4.1% of breast and of endometrial cancer samples. The reported frequency of *AKT1* mutations in breast cancer is between 1.4 and 8.2% [5, 6], and they are associated with histogenesis of hormone receptor positive subtype [15]. Thus, our finding that *AKT1* mutations are associated with HER2-negative status in breast cancer agrees with those of a previous study [15]. The TCGA (The Cancer Genome Atlas Program) data showed that in a predominantly Caucasian breast cancer cohort, *AKT1* mutations had a frequency of 3% in most hormone receptor positive breast cancers [16]. On the other hand, a report from China also showed that *AKT1* mutations were found in 7.1 and 8.2% of breast cancer patients [6, 17], suggesting that *AKT1* mutations may be particularly common in Asians.

Previous reports have suggested that activation of the PI3K/AKT pathway is involved in the development of endometrioid adenocarcinoma [7, 8, 18], and that atypical hyperplasia is also associated with *AKT1* mutations, leading to benign and (pre) malignant endometrial lesions [19]. In addition, *AKT1* mutations were found in atypical hyperplasia, suggesting that *AKT1* mutations may be involved in the carcinogenesis of endometrial carcinoma in benign and (pre) malignant endometrial lesions [19]. Louis J.M. van der Putten et al. reported We did not find a relationship between clinicopathological features and *AKT1* mutations because of the rarity of the mutation. However, none of the patients with mutated *AKT1* died. A longer follow-up might reveal a correlation between *AKT1* mutation status and its prognostic importance.

Our survival results themselves are difficult to say definitively due to the small number of cases. However, recently published results from the AACR Project GENIE, which incorporated a large group of estrogen receptor positive breast cancer patients, reported that *AKT1* mutant cases had comparable survival rates compared to *AKT1* wild-type controls [20].

Despite these positive findings, this study has three major limitations. The *AKT1* mutation is rare, and rates of relapse or death are extremely low. Therefore, we cannot reach any conclusions on the survival based on prognostic factors. Recently, with the development of next generation sequencing (NGS), cancer genomic profiling including *AKT1* are now being evaluated using NGS tests. It is an important limitation that this study only examined *AKT1* E17K hotspot mutation. Finally, there is fact that a large proportion of endometrial cancer is associated with a hypermutator phenotype, mainly due to MSI (microsatellite instability) and POLE (DNA polymerase epsilon) mutations. It is hard to distinguish the real *AKT1* driver mutations from random mutations caused by this phenotype.

Precision medicine is becoming important in the treatment of cancer, and the *AKT1* mutation is a promising target. Active point mutations (hot spots), are limited in breast and endometrial cancers, and E17K is a representative mutation site. The development of systems for detecting single mutations has progressed. Moreover, recent clinical trials have suggested that the *AKT1* mutation is a potent predictive marker of a response to AKT inhibitors [3, 4]. Therefore, the *AKT1* mutation might be a target of liquid biopsies, particularly in cell-free plasma DNA [21].

In conclusion, the rate of mutated *AKT1* in breast cancer was 7.4%, and it correlated with the HER2-negative subtype. The rate in endometrial cancer was 4.1%. *AKT1* mutations are associated with HER2-negative subtype in breast cancer and in endometrial cancer with endometrioid histology. The frequencies of *AKT1* mutations in breast and endometrial cancers were similar between Asian and other regional women. The frequency of mutations is too low in both tumor types to talk about predictive significance.

## Supplementary Information

**Additional file 1.**

**Additional file 2.**

## Data Availability

The datasets used and/or analysed during the current study are available from the corresponding author on reasonable request.

## Abbreviations

Akt: V-Akt murine thymoma viral oncogene
Bp: partial mastectomy
Bt: total mastectomy
ER: oestrogen receptor
FFPE: formalin-fixed paraffin-embedded
HER2: human epidermal growth factor receptor-2
HR: hormone receptor
IDC: invasive ductal carcinoma
ILC: invasive lobular carcinoma
LVI: lymphovascular invasion
mut: mutation
ND: no data
PgR: progesterone receptor
