# Supplementary material for: Hotspot mutation profiles of AKT1 in Asian women with breast and endometrial cancers
Source: BMC Cancer. 2021 Oct 21;21:1131. doi: 10.1186/s12885-021-08869-3 (PMC8529845; doi:10.1186/s12885-021-08869-3)
Supplement: Supplementary file 1 — Additional file 1. [file 12885_2021_8869_MOESM1_ESM.docx]

Additional Table 1. Cox hazard model in breast cancer (OS)

|  | HR | 95%CI | P-value |
| --- | --- | --- | --- |
| Stage III–IV  Stage I–II | 2.9  ref | 1.5-5.9 | 0.0020* |
| Grade 3  Grade 1–2 | 0.96  ref | 0.46–1.9 | 0.90 |
| HR-positive  HR-negative | 0.84  ref | 0.29–2.7 | 0.76 |
| HER2-positive  HER2-negative | 1.2  ref | 0.41–3.2 | 0.72 |
| *AKT1* mutation  Wild-type | 0.50  ref | 0.08–1.7 | 0.31 |

Multivariate analysis showed that the initial high stage was the sole poor prognostic factor for overall survival.

Additional Table 2. Cox hazard model in uterine endometrial cancer (OS)

|  | HR | 95% CI | P-value |
| --- | --- | --- | --- |
| Stage III–IV  Stage I–II | 5.3  ref | 2.2-14.2 | 0.00020* |
| Lymphovascular invasion-positive  Lymphovascular invasion-negative | 1.6  Ref | 0.57–5.7 | 0.31 |
| *AKT1* mutation  Wild-type | 8.8e^-10^  ref | 0.28–0.74 | 0.027* |

Multivariate analysis showed that the initial high stage and *AKT1* wild type were the poor prognostic factors for overall survival.
