# Supplementary material for: Hotspot mutation profiles of AKT1 in Asian women with breast and endometrial cancers
Source: BMC Cancer. 2021 Oct 21;21:1131. doi: 10.1186/s12885-021-08869-3 (PMC8529845; doi:10.1186/s12885-021-08869-3)
Supplement: Supplementary file 2 — Additional file 2. [file 12885_2021_8869_MOESM2_ESM.pdf]

Additional Figure 1.

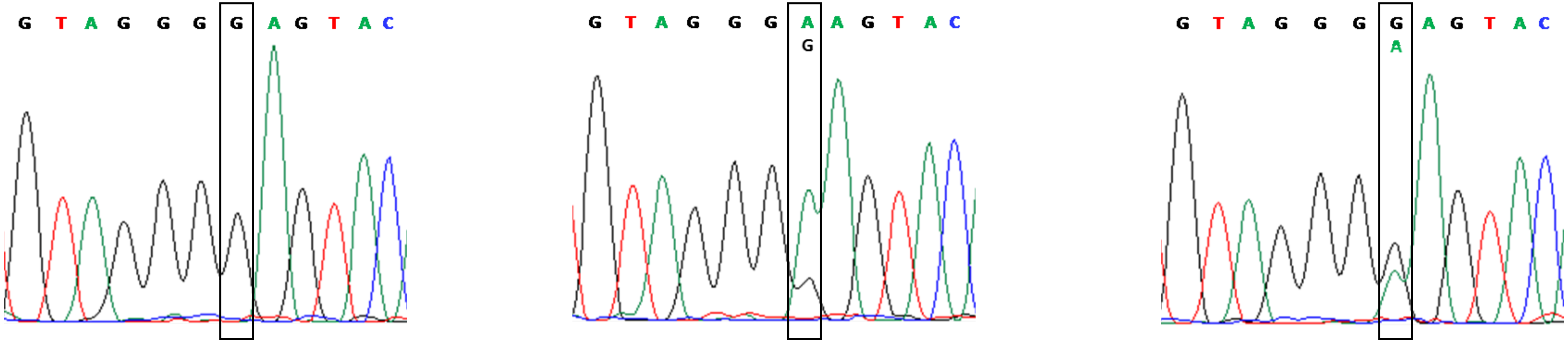

Tumour 1
AKT1 wild-type

Tumour 85
AKT1 (E17K) mutation-positive

Tumour 115
AKT1 (E17K) mutation-positive

Tumour 1, no *AKT1* mutations; Tumours 85 and 115, point mutation of G to A at nucleotide 49 (E17K).
